# Insights into the phylogenetic profile and virulence-associated genes of multidrug-resistant uropathogenic *Escherichia coli* in North of Iran

**DOI:** 10.1016/j.ijregi.2025.100711

**Published:** 2025-07-18

**Authors:** Raheleh Sheikhi, Sara Darvishvand, Mohammad Shenagari, Ali Monfared, Masoud Khosravi, Mojgan Sigarchi, Elham Ramezanzade, Mohammad-kazem Lebadi, Yalda Haghdar-Saheli, Pegah Aghajanzadeh, Ali Movassaghi

**Affiliations:** 1 Department of Microbiology, Virology and Microbial Toxins, School of Medicine, Guilan University of Medical Sciences, Rasht, Iran; 2 Urology Research Center, School of Medicine, Razi Hospital, Guilan University of Medical Sciences, Rasht, Iran

**Keywords:** Uropathogenic *Escherichia coli*, Virulence genes, Phylogenetic, Multi-drug resistant, Urinary tract infection

## Abstract

**Objectives:**

Urinary tract infections caused by multidrug-resistant uropathogenic *Escherichia coli* (UPEC) strains limit therapeutic options and pose a serious threat to global health. This study aimed to analyze the phylogenetic distribution and virulence genes of multidrug resistant (MDR) UPEC strains and their associated risk factors.

**Methods:**

UPEC isolates were subjected to phylogenetic and virulence genotyping using conventional and multiplex polymerase chain reaction methods. Antimicrobial susceptibility patterns were determined using disk diffusion method.

**Results:**

Among 105 UPEC isolates, phylogenetic group B2 was the most prevalent (42.7%), followed by unknown (21.3%), E (15.7%), B1 (13.5%), and D (6.7%) among MDR UPEC isolates. A high prevalence of virulence genes, including *hma* and *sitD* (98.9%), *fimH* (96.6%), *cirA* (92.1%), *fyuA* (89.9%), *iutA* and *sitC* (86.5%), *pgaC* and *kpsMTII* (68.5%), and *csgA* (67.4%), was observed across various phylogenetic groups of MDR UPEC isolates. Among MDR strains, the highest resistance rates were to ampicillin, ciprofloxacin, and sulfamethoxazole-trimethoprim. In contrast, the highest susceptibility, aside from carbapenems, was to fosfomycin, amikacin, cefoxitin, and nitrofurantoin. We identified that male gender may increase the risk of infection with MDR UPEC strains. The results of this study revealed that phylogenetic group B2 of UPEC isolates are associated with virulence-associated genes and antibiotic resistance more than other phylogenetic groups.

**Conclusions:**

The findings support the potential of these genes as targets for vaccine development and highlight the necessity for continued regional research to address antibiotic resistance and improve urinary tract infection management in order to select appropriate treatments.

## Highlights

• B2 was the most common uropathogenic *Escherichia coli* phylogroup, linked to virulence and resistance.
• Gene variability shows local differences, needing region-specific studies.
• Iron and adhesion genes are promising vaccine targets against urinary tract infection.
• Fosfomycin, nitrofurantoin, and carbapenems are effective for multidrug resistant urinary tract infections.

## Introduction

Urinary tract infections (UTIs) rank among the most prevalent infectious diseases and are predominantly caused by *Escherichia coli* (*E. coli*), specifically the uropathogenic *E. coli* (UPEC), which is implicated in 80–90% of community-acquired and 40–50% of hospital-acquired UTIs. UTIs are especially common in females across various age groups, with about 25% of these individuals experiencing recurrent infections [1,2]. *E. coli* strains are classified into eight phylogenetic groups: B2, B1, A, D, F, E, C, and clade I 3. Phylogenetic studies have revealed that the more virulent extraintestinal *E. coli* strains, which are frequently responsible for UTIs, primarily belong to phylogroup B2, with lesser representation from group D, while commensal strains are generally associated with phylogroups A or B1 [4]. UPEC expresses multiple surface-associated virulence factors, such as surface polysaccharide structures, adhesins, and iron acquisition systems, that enable them to adhere to uroepithelial cells, colonize the urinary tract, and contribute to pathogenesis, thus ensuring their persistence in the urinary system. Iron plays a crucial role in the survival and pathogenesis of UPEC strains, particularly in the urinary tract [5,6]. The ability of these bacteria to acquire iron from the host is mediated by various iron acquisition systems, which are regulated by the ferric uptake regulator (*fur*) system. In *E. coli* strains associated with UTIs, the presence of comprehensive iron acquisition systems is a well-documented feature compared to commensal *E. coli* strains [7]. Recent studies have emphasized the significance of these iron acquisition systems as potential targets for vaccine development aimed at preventing UTIs [8]. Previous studies have demonstrated that antibiotic resistance and the presence of virulence genes have been induced in certain *E. coli* strains, contributing to their virulence [1,9]. Excessive and inappropriate use of antibiotics has significantly contributed to the rising incidence of multidrug-resistant (MDR) strains. The emergence of MDR UPEC isolates has become a major challenge in the treatment of UTIs, leading to limited therapeutic options and posing a serious threat to global health [9]. In light of this, identifying the virulence-associated genes and risk factors contributing to antimicrobial resistance is essential for optimizing empirical treatment strategies for UTIs caused by MDR UPEC strains. Therefore, the present study sought to conduct a comparative analysis of the virulence-associated genes and phylogenetic profiles of MDR UPEC strains isolated from UTI patients, alongside an investigation of related risk factors. The findings are expected to provide valuable epidemiological insights and inform effective measures for the prevention, control, and management of UTIs in the studied region.

## Materials and methods

### Study design, sample collection and bacterial identification

This cross-sectional study was conducted from May 2019 to February 2020 at the Department of Microbiology, Virology, and Microbial Toxins, in collaboration with Razi Hospital, Rasht, Iran. The study was approved by the Research Ethics Committee of Guilan University of Medical Sciences (No. IR.GUMS.REC.1398.068). A total of 105 UPEC isolates were obtained from 130 UTI samples. The isolates were identified using standard bacteriological techniques, including colony morphology, gram staining, and conventional biochemical tests. Pure colonies were preserved at -80°C in trypticase soy broth with 20% glycerol for further analysis.

### Antimicrobial susceptibility testing

The antibiotic susceptibility of UPEC isolates was assessed using the disk diffusion method (Oxoid Limited, United Kingdom) on Mueller-Hinton agar against the following antibiotics: ampicillin (AM; 10 µg), ampicillin-sulbactam (SAM; 10/10 µg), amoxicillin-clavulanate (AMC; 20/10 µg), piperacillin- tazobactam (PTZ; 100/10 µg), cefazolin (CZ; 30 µg), cefuroxime (CXM; 30 µg), ceftriaxone (CRO; 30 µg), cefepime (CPM; 30 µg), cefoxitin (FOX; 30 µg), imipenem (IMP; 10 µg), meropenem (MEM; 10 µg), gentamicin (GM; 10 µg), amikacin (AK; 30 µg), ciprofloxacin (CIP; 5 µg), levofloxacin (LEV; 5 µg), trimethoprim-sulfamethoxazole (TS; 1.25/23.75 µg), nitrofurantoin (F/M; 300 μg), fosfomycin (FOS; 200 µg), and azithromycin (AZM; 15 µg) according to the guidelines of the Clinical and Laboratory Standards Institute (CLSI), 2021 [10]. Isolates were considered MDR if resistant to at least one agent in three or more antibiotics classes [11].

### Phylogenetic classification and virulence genotyping

Chromosomal DNA of all isolated UPEC strains was extracted using the boiling method, as previously described [12]. Phylogenetic classification of UPEC strains was conducted using quadruplex polymerase chain reaction (PCR) phylo-typing with primers and conditions described by Clermont et al [2,4]. Conventional and multiplex PCR were performed to amplify various adhesin and iron acquisition genes using specific primers (Table 1). Amplified products were analyzed by electrophoresis on a 1.5% agarose gel. Multiplex PCRs were carried out to amplify different iron uptake genes, as listed below:

**Table 1:** List of primers used in this study.

Genes	Function	Primers (5′-3′)	Tm (°C)	Ref.
***fimH***	Type 1 fimbrial	F-TGCAGAACGGATAAGCCGTGG R-GCAGTCACCTGCCCTCCGGTA	67.6	[5]
***papC***	P fimbrial	F-GTGGCAGTATGAGTAATGACCGTTA R-ATATCCTTTCTGCAGGGATGCAATA	60	
***papGII***		F-GGGATGAGCGGGCCTTTGAT R-CGGGCCCCCAAGTAACTCG	64	
***afa/draBC***	Mannose-resistant hemagglutination	F-GGCAGAGGGCCGGCAACAGGC R-CCCGTAACGCGCCAGCATCTC	67	
***kpsMTII***	Polysaccharide capsule	F-GCGCATTTGCTGATACTGTTG R-CATCCAGACGATAAGCATGAGCA	59	
***flu (agn43)***	cell-to-cell aggregation	F-ACGCACAACCATCAATAAAA R-CCGCCTCCGATACTGAATGC	52	[25]
***pgaC***	Responsible for production of β-1,6-N-acetyl-D-glucosamine	F-ATGATTAATCGCATCGTATCG R-CATCGGTTCCACAATATATGC	60	[26]
***csgA***	Regulators the expression of the curli genes	F-ATCTGACCCAACGTGGCTTCG R-GATGAGCGGTCGCGTTGTTACC	61	[27]
***fyuA***	Iron uptake receptor	F-TGATTAACCCCGCGACGGGAA R-CGCAGTAGGCACGATGTTGTA	63	[28]
***irp2***	Siderophore receptors	F-AAGGATTCGCTGTTACCGGAC R-TCGTCGGGCAGCGTTTCTTCT	59	
***iutA***		F-GGCTGGACATCATGGGAACTGG R-CGTCGGGAACGGGTAGAATCG	63	
***iroN***		F-AAGTCAAAGCAGGGGTTGCCCG R-GACGCCGACATTAAGACGCAG	63	
***fur***	Ferric uptake regulator	F-TTTAGGCGTGGCAATTCTATAATGA R-TATCAGTCATGCGGAATCTGTCCTG	60	[7]
***hma***	Iron transporters	F-ATGGTTAAAGATACAATC R-CCACTGATAACGGGTAT	56	[6]
***sitA***		F-AGGGGGCACAACTGATTCTCG R-TACCGGGCCGTTTTCTGTGC	59	[28]
***sitD***		F-CTGTGCGCTGCTGTCGGTC R-GCGTTGTGTCAGGAGTAC	52	[29]
***mntH***		F-CTAAGCTTCGTAGGGCGGATAAGGCGTT R-GGTTAAGCTTCCGTGCACATTCTATGTAA	64	[30]

#### Triplex PCR for sitA, irp2 and kpsMTII genes

A 20 µl reaction consisted of 10 µl of master mix, one µl each of forward and reverse primers (10 pmol/µl), 5 µl of sterile water, and 1 µl of DNA. The thermal cycling process began with initial denaturation at 94°C for 5 minutes, followed by 30 cycles of denaturation at 94°C for 30 seconds, annealing at 59°C for 30 seconds, and extension at 72°C for 30 seconds, with a final extension at 72°C for 5 minutes.

#### Triplex PCR for fyuA, iroN and iutA genes

A 20 µl reaction consisted of 10 µl of master mix, 1 µl each of forward and reverse primers (10 pmol/µl), 5 µl of sterile water, and 1 µl of DNA. The thermal cycling process began with initial denaturation at 95°C for 5 minutes, followed by 25 cycles of denaturation at 94°C for 30 seconds, annealing at 63°C for 30 seconds, and extension at 72°C for 5 minutes, with a final extension at 72°C for 10 minutes.

#### Triplex PCR targeting fur, papC, and pgaC

The reaction mixture included 10 µl of master mix, 1 µl each of forward and reverse primers (10 pmol/µl), 5 µl of sterile water, and 1 µl of DNA, totaling 20 µl. The process started with a 5-minute denaturation at 95 °C, followed by 35 cycles of denaturation at 95°C for 15 seconds, annealing at 60°C for 15 seconds, and extension at 72°C for 1 minute, finishing with a 5-minute extension at 72°C.

#### Duplex PCR for papGII and mntH genes

A 20 µl reaction consisted of 10 µl of master mix, 1 µl each of forward and reverse primers (10 pmol/µl), 5 µl of sterile water, and 1 µl of DNA. The thermal cycling process began with initial denaturation at 94°C for 5 minutes, followed by 30 cycles of denaturation at 94°C for 30 seconds, annealing at 64°C for 30 seconds, and extension at 72°C for 30 seconds, with a final extension at 72°C for 5 minutes.

#### Duplex PCR for sitD and flu genes

A 20 µl reaction included 10 µl of master mix, 1 µl each of forward and reverse primers (10 pmol/µl), 5 µl of sterile water, and 1 µl of DNA. The thermal cycling process began with initial denaturation at 94°C for 5 minutes, followed by 30 cycles of denaturation at 94°C for 30 seconds, annealing at 52°C for 15 seconds, and extension at 72°C for 30 seconds, with a final extension at 72°C for 5 minutes.

A conventional PCR was performed to amplify *fimH, afa/draBC, csgA* and *hma* genes. The amplification conditions were as follows: denaturation at 94°C for 3 minutes; 30 cycles of denaturation at 94°C for 1 minute, annealing at specific temperature (Table 1) for 1 minute, and extension at 72°C for 3 minutes; and a final extension at 72°C for 5 minutes.

### Statistical analysis

Data were analyzed using SPSS™ software, version 21.0. Descriptive statistics were used to present results, and chi-square (χ²) tests evaluated the relationships between variables. Statistical significance was set at *P* ≤ 0.05.

## Results

A total number of 105 UPEC strains were collected from patients with UTI, predominantly from females (84.8%). The majority of the patients belonged to the age group 61–80 years (53%).

### Phylogenetic group distribution of UPEC strains

In this study, UPEC isolates were analyzed according to new Clermont Quadruplex PCR method, resulting in the classification of these isolates into five phylogenetic groups. Phylo-typing results showed that phylogenetic group B2 was the most prevalent, 38 (42.7%), followed by group unknown, 19 (21.3%); group E, 14 (15.7%); group B1, 13 (13.5%); and group D, 6 (6.7%) among MDR UPEC isolates.

### Antibiotic resistance profiles of UPEC strains

Antibiotic resistance of the UPEC isolates was investigated using 10 classes of antibiotics. Out of 105 UPEC isolates, 89 (84.8%) were MDR. The antibiotic resistance pattern was compared between MDR and non-MDR strains (Table 2). The MDR strains shown significantly (*P* < 0.05) higher degree of resistance to all of the studied antibiotics except FOX, AK, IMP, MEM, F/M, and FOS, compared to non-MDR strains. Among MDR strains, the highest resistance rate was observed against AM (95.5%), followed by CIP (86.5%), and LEV and TS (77.5%). However, the highest susceptibility was toward carbapenems such as IMP (96.6%) and MEM (94.4%), followed by FOS (91%) and AK (87.6%). The incidence of antibiotic resistance in different phylogenetic groups of MDR UPEC isolates is shown in Table 3. According to our results, there was no significant correlation between phylogenetic groups and antibiotic resistance, except for resistance to AM, CXM, CPM, CRO and AZM, which was higher in phylogroups B2, unknown, and E.

**Table 2:** Antibiotic resistance pattern of MDR uropathogenic *Escherichia coli* isolates.

Antibiotics	MDR (n = 89)		No-MDR (16)		*P*-value
	Resistant	Susceptible	Resistant	Susceptible	
AM	85(95.5)	4(4.5)	9(56.2)	7(43.8)	0.000
SAM	55(61.8)	34(38.2)	3(18.8)	13(81.2)	0.001
AMC	56(62.9)	33(37.1)	3(18.8)	13(81.2)	0.001
PTZ	28(31.5)	61(68.5)	0(0)	16(100)	0.009
CZ	56(62.9)	33(37.1)	0(0)	16(100)	0.001
CXM	67(75.3)	22(24.7)	0(0)	16(100)	0.001
CPM	57(64)	32(36)	1(6.2)	15(93.8)	0.001
CRO	46(51.7)	43(48.3)	0(0)	16(100)	0.001
FOX	16(18)	73(82)	0(0)	16(100)	0.06
GM	28(31.5)	61(68.5)	0(0)	16(100)	0.009
AK	11(12.4)	78(87.6)	0(0)	16(100)	0.1
CIP	77(86.5)	12(13.5)	2(12.5)	14(87.5)	0.001
LEV	69(77.5)	20(22.5)	1(6.2)	15(93.8)	0.001
T/S	69(77.5)	20(22.5)	3(18.8)	13(81.2)	0.001
IMP	3(3.4)	86(96.6)	0(0)	16(100)	0.4
MEM	5(5.6)	84(94.4)	0(0)	16(100)	0.3
F/M	21(23.6)	68(76.4)	1(6.2)	15(93.8)	0.1
FOS	8(9)	81(91)	1(6.2)	15(93.8)	0.7
AZM	52(58.4)	37(41.6)	0(0)	16(100)	0.001

AM, ampicillin; AK, amikacin; AMC, amoxicillin-clavulanate; AZM, azithromycin; CIP, ciprofloxacin; CPM, cefepime; CRO, ceftriaxone; CZ, cefazolin; CXM, cefuroxime; F/M, nitrofurantoin; FOS, fosfomycin; FOX, cefoxitin; GM, gentamicin; IMP, imipenem; LEV, levofloxacin; MDR, multidrug resistant; MEM, meropenem; PTZ, piperacillin-tazobactam; TS, trimethoprim-sulfamethoxazole; SAM, ampicillin-sulbactam.

**Table 3:** Distribution of antibiotic resistance and virulence genes among phylogenetic groups of multidrug resistant uropathogenic *Escherichia coli* isolates.

Resistance traits	Total (n = 89) n(%)	Group UK^a^ (n = 19) n(%)	Group B1 (n = 12) n(%)	Group B2 (n = 38) n(%)	Group E (n = 14) n (%)	Group D (n = 6) n (%)	*P*-value
AM	85(95.5)	19(100)	11(91.7)	38(100)	11(78.6)	6(100)	0.01
SAM	55(61.8)	9(47.4)	8(66.7)	26(68.4)	10(71.4)	2(33.3)	0.27
AMC	56(62.9)	11(57.9)	7(58.3)	28(73.7)	8(57.1)	2(33.3)	0.32
PTZ	28(31.5)	7(36.8)	1(8.3)	15(39.5)	4(28.6)	1(16.7)	0.28
CZ	56(62.9)	12(63.2)	6(50)	27(71.1)	9(64.3)	2(33.3)	0.38
CXM	67(75.3)	16(84.2)	5(41.7)	34(89.5)	9(64.3)	3(50)	0.00
CPM	57(64)	14(73.7)	3(25)	30(78.9)	7(50)	3(50)	0.00
CRO	46(51.7)	11(57.9)	3(25)	25(65.8)	6(42.9)	1(16.7)	0.03
FOX	16(18)	5(26.3)	3(25)	4(10.5)	2(14.3)	2(33.3)	0.43
GM	28(31.5)	8(42.1)	2(16.7)	14(36.8)	1(7.1)	3(50)	0.11
AK	11(12.4)	0(0)	2(16.7)	5(13.2)	3(21.4)	1(16.7)	0.39
CIP	77(86.5)	15(78.9)	10(83.3)	35(92.1)	12(85.7)	5(83.3)	0.71
LEV	69(77.5)	14(73.7)	7(58.3)	34(89.5)	9(64.3)	5(83.3)	0.11
T/S	69(77.5)	12(63.2)	11(91.7)	31(81.6)	10(71.4)	5(83.3)	0.35
IMP	3(3.4)	0(0)	1(8.3)	1(2.6)	0(0)	1(16.7)	0.25
MEM	5(5.6)	1(5.3)	2(16.7)	0(0)	2(14.3)	0(0)	0.11
F/M	21(23.6)	4(21.1)	4(33.3)	10(26.3)	3(21.4)	0(0)	0.6
FOS	8(9)	1(5.3)	1(8.3)	2(5.3)	3(21.4)	1(16.7)	0.39
AZM	52(58.4)	8(42.1)	2(16.7)	27(71.1)	10(71.4)	5(83.3)	0.00

**Virulence factors**							

*fimH*	86(96.6)	17(89.5)	12(100)	38(100)	14(100)	5(83.3)	0.07
*papC*	41(46.1)	4(21.1)	6(50)	22(57.9)	6(42.9)	3(50)	0.13
*papGII*	45(50.6)	7(36.8)	5(41.7)	22(57.9)	7(50)	4(66.7)	0.51
*afa/draBC*	46(51.7)	9(47.4)	7(58.3)	17(44.7)	9(64.3)	4(66.7)	0.64
*kpsMTII*	61(68.5)	10(52.6)	8(66.7)	29(76.3)	9(64.3)	5(83.3)	0.4
*flu (agn43)*	46(51.7)	10(52.6)	5(41.7)	23(60.5)	5(35.7)	3(50)	0.53
*pgaC*	61(68.5)	14(73.7)	6(50)	27(71.1)	10(71.4)	4(66.7)	0.67
*csgA*	60(67.4)	12(63.2)	8(66.7)	24(63.2)	11(78.6)	5(83.3)	0.74
*iroN*	51(57.3)	12(63.2)	8(66.7)	21(55.3)	7(50)	3(50)	0.87
*fyuA*	80(89.9)	18(94.7)	11(91.7)	36(94.7)	10(71.4)	5(83.3)	0.13
*irp2*	58(65.2)	11(57.9)	9(75)	27(71.1)	8(57.1)	3(50)	0.63
*iutA*	77(86.5)	14(73.7)	10(83.3)	34(89.5)	13(92.9)	6(100)	0.34
*mntH*	59(66.3)	7(36.8)	5(41.7)	34(89.5)	10(71.4)	3(50)	0.00
*fur*	66(74.2)	12(63.2)	10(83.3)	35(92.1)	7(50)	2(33.3)	0.00
*hma*	88(98.9)	19(100)	12(100)	38(100)	13(92.9)	6(100)	0.24
*sitA*	46(51.7)	3(15.8)	6(50)	31(81.6)	4(28.6)	2(33.3)	0.00
*sitD*	88(98.9)	18(94.7)	12(100)	38(100)	14(100)	6(100)	0.44

a UK; unknown AM, ampicillin; AK, amikacin; AMC, amoxicillin-clavulanate; AZM, azithromycin; CIP, ciprofloxacin; CPM, cefepime; CRO, ceftriaxone; CXM, cefuroxime; CZ, cefazolin; F/M; Nitrofurantoin, FOS; Fosfomycin, FOX, cefoxitin; GM, gentamicin; IMP, imipenem; LEV, levofloxacin; MEM, meropenem; PTZ, piperacillin-tazobactam; SAM, ampicillin-sulbactam; TS, trimethoprim-sulfamethoxazole.

### Virulence genotyping profiles of UPEC strains

A total of 21 different virulence factor genes related to adhesins and iron acquisition systems were evaluated among 105 UPEC isolates, as presented in Table 4. Genotyping analysis revealed that the *fimH* (96.6%) was the most prevalent adhesin gene, followed by *pgaC* and *kpsMTII* (68.5%), and *csgA* (67.4%). Also, *hma* and *sitD* (98.9%) were the most prevalent iron acquisition genes, followed by *fyuA* (89.9%) and *iutA* (86.5 %) among MDR compared to non-MDR isolates. Overall, no significant difference was observed in the distribution of virulence genes between MDR and non-MDR UPEC isolates. The prevalence of virulence genes in various phylogenetic groups (Table 3) showed that there was no significant correlation between the phylogenetic groups and studied virulence genes of MDR UPEC strains, except *mntH, fur* and *sitA*, which were most prevalent in phylogroups B2. Overall, the prevalence of iron acquisition genes was high across most phylogenetic groups, with the highest average frequencies observed in groups B2 and D.

**Table 4:** Distribution of virulence genes among MDR and non-MDR of uropathogenic *Escherichia coli* isolates.

	MDR (89) no. (%)	No-MDR (16) no. (%)	*P*-value
**Adhesin genes**			
*fimH*			0.45
Positive	86(96.6)	16(100)	
Negative	3(3.4)	0(0)	
*papC*			0.86
Positive	41(46.1)	7(43.8)	
Negative	48(53.9)	9(56.2)	
*papGII*			0.67
Positive	45(50.6)	9(56.2)	
Negative	44(49.4)	7(43.8)	
*flu (agn43)*			0.01
Positive	46(51.7)	3(18.8)	
Negative	43(48.3)	13(81.2)	
*pgaC*			0.05
Positive	61(68.5)	7(43.8)	
Negative	28(31.5)	9(56.2)	
*afa/draBC*			0.2
Positive	46(51.7)	11(68.8)	
Negative	43(48.3)	5(31.2)	
*kpsMTII*			0.98
Positive	61(68.5)	11(68.8)	
Negative	28(31.5)	5(31.2)	
*csgA*			0.07
Positive	60(67.4)	7(43.8)	
Negative	29(32.6)	9(56.2)	
**Iron acquisition genes**			
*fyuA*			0.31
Positive	80(89.9)	13(81.2)	
Negative	9(10.1)	3(18.8)	
*iroN*			0.31
Positive	51(57.3)	7(43.8)	
Negative	38(42.7)	9(56.2)	
*irp2*			0.07
Positive	58(65.2)	14(87.5)	
Negative	31(34.8)	2(12.5)	
*iutA*			0.57
Positive	77(86.5)	13(81.2)	
Negative	12(13.5)	3(18.8)	
*fur*			0.33
Positive	66(74.2)	10(62.5)	
Negative	23(25.8)	6(37.5)	
*hma*			0.67
Positive	88(98.9)	16(100)	
Negative	1(1.1)	0(0)	
*sitA*			0.9
Positive	46(51.7)	8(50)	
Negative	43(48.3)	8(50)	
*sitD*			0.67
Positive	88(98.9)	16(100)	
Negative	1(1.1)	0(0)	
*mntH*			0.76
Positive	59(66.3)	10(62.5)	
Negative	30(33.7)	6(37.5)	

MDR, multidrug resistant.

### Risk factors for MDR UPEC UTIs

In our study, analysis of the risk factors for MDR strains showed that gender might predispose patients to infection by MDR UPEC strains (*P* = 0.05) (Table 5).

**Table 5:** Risk factors and demographic characteristics of patients for infection with MDR uropathogenic *Escherichia coli* strains.

Risk factors	MDR (+) (n = 89)	MDR (-) (n = 16)	*P*-value
Male gender	16(18)	0(0)	0.05
Female gender	73(82)	16(100)	
Age >60 years	55(63.2)	6(46.2)	0.23
Age <60 years	32(36.8)	7(53.8)	
Recurrent urine tract infection			
Yes	71(81.6)	9(69.2)	0.29
No	16(18.4)	4(30.8)	
History of antibiotic use in last 3 months			
Yes	63(72.4)	10(76.9)	0.7
No	24(27.6)	3(23.1)	
History of hospitalization in last 3 months			
Yes	44(50.6)	5(38.5)	0.4
No	43(49.4)	8(61.5)	
History of urinary catheterization			
Yes	20(23)	2(15.4)	0.5
No	67(77)	11(84.6)	
Hypertension			
Yes	57(65.5)	7(53.8)	0.4
No	30(34.5)	6(46.2)	
Urinary tract stone			
Yes	12(13.8)	1(7.7)	0.5
No	75(86.2)	12(92.3)	
Diabetes mellitus			
Yes	31(35.6)	4(30.8)	0.7
No	56(64.4)	9(69.2)	

MDR; multidrug resistant.

## Discussion

UPEC is the predominant etiological agent of UTIs, a disease of major significance to global human health [8]. The virulence-associated genes play important roles in the pathogenicity and development of extraintestinal *E. coli* infections, so the virulence content in pathogenic and commensal strains usually varies and is typically related to the phylogenetic groups [9,13]. Development of novel therapeutic or prophylactic strategies, such as vaccine development against UPEC strains, requires a more comprehensive understanding of virulence determinants, antibiotic resistance, and their relationship with each other. Also, the distribution of pathogenic factors and their relation to phylogenetic groups indicate a complex interplay between genetic background and pathogenic behavior. Therefore, this trend underscores the clinical relevance of assigning isolates to specific phylogenetic groups, which could enhance the understanding of infection mechanisms and help identify new targets for intervention in UTIs.

Out of 105 UPEC isolates, the prevalence of MDR isolates (84.8%) was higher than the pooled prevalence of MDR UPEC with 65.8% in Iran [14]. Our results demonstrated that not only were the majority of MDR isolates susceptible to IMP, MEM, and FOS but also susceptibility to AK, FOX, and F/M was also considerable, which was consistent with some previous reports from Iran and other countries [1,13]. To prevent the increase of carbapenem-resistant strains, the use of alternative antibiotics is essential. Thus, considering the recommendation to avoid using carbapenems as first-line treatment for infections caused by MDR Enterobacterales, our results suggest that oral treatment options for UTIs due to drug-resistant strains in our study region include F/M and FOS. In agreement with previous studies, in this study, based on statistical analysis, there was no significant difference in the phylogenetic group composition with respect to antibiotic resistance [15], except that the resistance patterns for AM, CXM, CPM, CRO and AZM indicated that these isolates were mostly in phylogroups B2, unknown and E. Overall, according to our results, phylogenetic group B2 is the most drug resistant, similar to other published reports [2,16].

The current study is notable for its investigation of UPEC isolates from UTIs based on the phylogenetic classification system [4]. Consistent with previous studies in Iran and other countries, our results showed that among 89 MDR UPEC isolates, the majority (42.7%) belonged to phylogroup B2 [2,15,17,18]. Also, our findings were not consistent with those of Farajzadeh Sheikh et al [9], whose study on UPEC phylogroups, showed the highest prevalence in phylogroup D. In our study, phylogroup A—associated with commensal *E. coli* strains—was not detected; similarly, in another study, phylogroup A had the lowest prevalence [2]. In agreement with some previous studies [2], our results showed that the prevalence of phylogroups D and B1 was much lower than in other countries [19,20]. This finding aligns with global studies but exhibits regional variations, highlighting the influence of geographic and health factors on UPEC strain phylogenetic distributions [21].

UPEC strains encoded a variety of adhesin-associated genes such as fimbrial adhesins (e.g., *fimH, papC*, and *papGII*), afimbrial adhesins (*afa/draBC*), and polysaccharide surface structures (*kpsMTII*), which allow them to colonize and survive in the urinary tract [12]. In agreement with some previous studies, our results showed that the distribution of adhesin genes exhibited similar frequencies across all phylogroups [15,17]. Several previous studies, including this one, reported a high frequency of *fimH* (94.5%) in various phylogenetic groups [9]. Also, our results showed that frequency of *flu* and *pgaC* was higher in pathogenic strains compared to commensal strains.

A critical aspect of this study was the examination of iron acquisition gene frequencies across different phylogenetic groups. Previous research has demonstrated that iron acquisition genes are more prevalent in extraintestinal pathogenic strains compared to commensal ones, correlating with increased virulence [8]. Our findings, however, showed that among iron acquisition genes—except for three genes including *mntH, fur* and *sitA*, which were dominant in pathogenic phylogroup B2 compared to commensal phylogroup B1—the distribution of other iron acquisition genes was relatively uniform across all phylogenetic groups, suggesting horizontal gene transfer between pathogenic and commensal strains [22]. The study also observed that the distribution of certain genes, like *mntH* and *sitA*, was higher in phylogroup B2 compared to commensal pyhlogroup B1. These genes are critical for iron acquisition and pathogenicity. Interestingly, some iron acquisition genes, such as *sitD, hma, irp2, fyuA, iutA* and *iroN*, were present at similar frequencies in both pathogenic and commensal strains, suggesting these genes are conserved across different *E. coli* phylogroups. Conversely, the *fur* gene, which regulates iron homeostasis, displayed varying frequencies, with higher prevalence in phylogroup B2 compared to other phylogroups. This variation might indicate different regulatory mechanisms affecting virulence and iron acquisition [22]. Thus, overall, the results of this study revealed that phylogenetic group B2 of UPEC isolates is associated with virulence-associated genes and resistant to antibiotics rather than other phylogenetic groups.

UTIs seen in men are considered mostly complicated, leading to long-term antibiotic use and increasing the incidence of MDR bacteria in this gender. In agreement with a previous study, our results showed that male gender might be a risk factor for infection with MDR UPEC strains [23]. Other previous studies have also shown older age, history of antibiotic use in the last three months, urinary catheterization, previous hospitalization, diabetes mellitus, and recurrent UTI [23,24] as predisposing factors that increase the risk of UTIs by MDR bacteria.

This study has several limitations. It was conducted in a single center with a relatively small sample size of 105 isolates, which may limit the generalizability of the findings. Additionally, the study did not evaluate the expression levels of detected virulence genes, restricting insights into their functional role. Important patient-related risk factors, such as previous antibiotic usage and comorbidities, were not thoroughly analyzed due to sample size constraints. Future research involving larger, multicenter studies and functional analyses of gene expression is recommended to validate and expand upon these findings.

## Conclusion

The findings from this study underscore that phylogenetic group B2 in UPEC isolates from UTIs is associated with virulence-associated genes and resistant to antibiotics, rather than other phylogenetic groups in the studied region. The distribution of adhesins and iron acquisition genes, which are critical for bacterial pathogenicity, was relatively similar across different phylogenetic groups of MDR UPEC isolates, suggesting that these genes are integral to both pathogenic and commensal *E. coli* strains. The variability in gene frequencies observed aligns with global studies but also highlights the need for region-specific research. We identified that male gender might be a risk factor for infection with MDR strains in our studied region. Our findings revealed that, in addition to carbapenems, oral therapeutic options including FOS and F/M are recommended for treating MDR strains in our studied region. Given the rising concern of antibiotic resistance and the significant health burden of UTIs, identifying iron acquisition genes as potential vaccine targets offers a promising avenue for prevention strategies. Future research should focus on detailed expression studies and animal models to elucidate the precise role of these genes in bacterial pathogenesis. This approach could lead to the development of effective vaccines and new strategies for combating UTIs caused by *E. coli*.

## Funding

This work was supported by the Vice Chancellor of Research and Technology, Guilan University of Medical Sciences, Rasht, Iran (Grant number: 98020202).

## Ethical approval statement

This study has been approved by the Research Ethics Committee (No. IR.GUMS.REC.1398.068), the Vice Chancellor of Research and Technology, Guilan University of Medical Sciences, Rasht, Iran.

## Author contributions

**Raheleh Sheikhi:** Conceptualization; formal analysis; investigation; methodology; supervision; writing – original draft; writing – review & editing. **Sara Darvishvand:** Methodology. **Mohammad Shenagari:** Methodology; writing – original draft. **Ali Monfared:** Methodology. **Masoud Khosravi:** Methodology. **Mojgan Sigarchi:** Methodology. **Elham Ramezanzade:** Methodology. **Mohammad-kazem Lebadi:** Methodology. **Yalda Haghdar-Saheli:** Methodology. **Pegah Aghajanzadeh:** Methodology. **Ali Movassaghi:** Methodology. All authors read and approved the final manuscript.

## Declaration of competing interest

The authors have no competing interests to declare.
